# Atypical periodic alternating nystagmus responding to high-dose intravenous immunoglobulins: a case report

**DOI:** 10.1186/s12974-017-0846-1

**Published:** 2017-03-31

**Authors:** Herminia Argente-Escrig, Luis Bataller, Claudio Krstulovic Roa, Vanesa Pérez Guillén, Herminio Perez Garrigues, Bonaventura Casanova Estruch

**Affiliations:** 1 Department of Neurology, Hospital Universitari i Politècnic La Fe, 106 Fernando Abril Martorell Ave, 46026 Valencia, Spain; 2 Department of Otolaryngology, Hospital Universitari i Politècnic La Fe, Valencia, Spain

**Keywords:** Nystagmus, Oscillopsia, Autoimmune disease, Gait disorders, Ataxia, Cerebellum

## Abstract

**Background:**

Acquired periodic alternating nystagmus (PAN) is a rare but well-defined syndrome that consists of a horizontal nystagmus that cyclically reverses its direction. PAN can be caused by degenerative, neoplastic, or toxic diseases of the cerebellum and, in a few cases, by subacute cerebellar ataxia of immune origin.

**Case presentation:**

A 44-year-old man came to our attention because of rapidly progressive gait instability and blurred vision. Clinical examination showed PAN and a mild pancerebellar syndrome. Eye movement recordings disclosed a short cycle PAN with significant slow-phase velocity only in darkness. Under the effect of a γ-aminobutyric acid type B (GABA_B_) agonist, PAN was not modified. Right after treatment with intravenous immunoglobulin (IVIg) was started, PAN was essentially eliminated. Three months after last dose of IVIg, this nystagmus reappeared.

**Conclusions:**

IVIg resolved PAN in this patient. This finding may point to an autoimmune mechanism underlying this patient’s nystagmus. This case suggests that the usefulness of IVIg at treating PAN might be worth a consideration in similar clinical settings.

## Background

Acquired periodic alternating nystagmus (PAN) is a rare but well-defined syndrome that consists of a horizontal nystagmus that cyclically reverses its direction. The duration of cycles typically lasts 2 min [1], but shorter intervals have been reported [2]. Several case reports indicate that there might be a good response to GABA_B_-ergic medications [3]. PAN has been reported in the context of degenerative, neoplastic, or toxic diseases of the cerebellum and occasionally in subacute cerebellar ataxia of presumed autoimmune origin [4].

## Case presentation

**Fig. 1:**
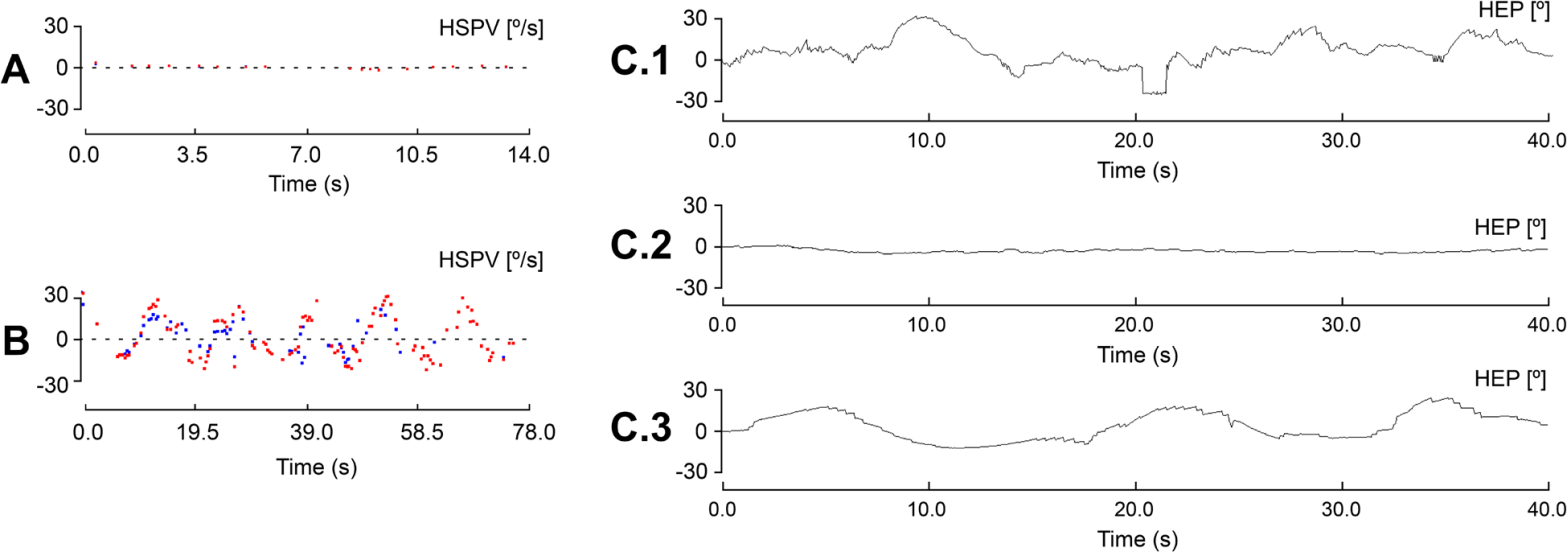
Videonystagmographic recordings of a patient with periodic alternating nystagmus responsive to intravenous immunoglobulins. **a** Horizontal slow-phase velocity (HSPV) recording at admission showed a maximum speed of 6.3°/s in light. **b** HSPV recording at admission exhibited a maximum speed of 30.7°/s in the dark. **c.1**–**c.3** Horizontal eye position (HEP) for each eye movement is plotted against time in the dark. **c.1** Under the effect of clonazepam alone showed PAN with a periodicity of 12 s comprised of 5 s of right-beating nystagmus and a 2-s steady phase followed by 5 s of left-beating nystagmus. **c.2** During the use of clonazepam and intravenous immunoglobulins, in which no nystagmus is observed. **c.3** During the use of clonazepam and 3 months after the last dose of intravenous immunoglobulins, PAN appeared again with similar characteristics as in **c.1**

A 44-year-old male patient with rapidly progressive gait difficulties, blurred vision, and tremor of 6 months’ duration has been studied. His past medical history was significant for human immunodeficiency virus (HIV) infection and intravenous heroin use 14 years prior to presentation. He also admitted that he occasionally used cannabis and amphetamines, but they were used prior to starting heroin. There was no history of head trauma. He was on highly active antiretroviral therapy (efavirenz, emtricitabine, and tenofovir) and methadone. Neurological examination revealed saccadic smooth pursuit, abnormal eye movements suggestive of PAN, dysarthria, head tremor as a “yes-yes” motion, and symmetric cerebellar tremor. There was mild dysmetria on finger-nose testing on the left side of the body. While resting or in action, he showed non-rhythmic myoclonic jerks involving arm muscles. Stance was broad-based without Romberg sign, and gait was ataxic. Pinprick sensation and vibration sense were mentioned as normal by the patient. Neither pyramidal nor parkinsonian signs were present. Videonystamographic recordings (Fig. 1A, B) showed a short cycle PAN only apparent in the dark whose peak slow-phase velocity was approximately 6°/s in light (Fig. 1A) and increased up to 30°/s in darkness (Fig. 1B).

Brain magnetic resonance imaging (MRI) showed no abnormalities, not even cerebellar atrophy. Comprehensive serum and blood laboratory investigations were normal and included vitamin E levels, thyroid function, antinuclear antibodies, and extensive serologies including those of hepatitis C and B virus and syphilis (all negative). He had a CD4+ count of 552 cells per cubic millimeter. Cerebrospinal fluid (CSF) examination showed lymphocytic predominant pleocytosis (37/μL), high protein concentration (115 mg/dl, normal <40 mg/dl), and absence of oligoclonal bands. Microbiological CSF studies were negative; they included HIV (fully suppressed HIV viral load with fewer than 20 copies/ml in the CSF) and John Cunningham (JC) virus polymerase chain reaction (PCR), the latter being negative in two measurements. A whole-body [^18^F]6FDG positron emission tomography (PET) scan did not reveal an occult neoplasm. Classic onconeuronal, anti-neuropil, and glutamic acid decarboxylase (GAD Ab) 65 antibodies were negative on serum and CSF measured with immunohistochemistry as described in a recent practice guide [5]. At the same time, GAD Ab 65 was also measured on sera with a commercial enzyme-linked immunosorbent assay (ELISA) kit (ElisaRSR™ GADAb) and was not detected.

The patient was started on clonazepam (at a dose of 1 mg every 8 h). Tremor slightly improved but PAN remained unchanged (Fig. 1C.1). A trial with high-dose IVIg (Flebogamma® produced by Grifols), at a dose of 17.5 g per day during 4 days for a weight of 70 kg, resulted in a dramatic improvement of the abnormal eye movements as PAN completely resolved (Fig. 1C.2).

During his hospital admission, the patient developed biopsy proven cutaneous lymphocytic vasculitis, acute severe autoimmune thrombocytopenia, and type IA diabetes. At this point, antibodies against gliadin and gangliosides GM1, GM2, and GD1 were determined but were not detected. Only after diabetes mellitus was diagnosed, levels of GAD Ab 65 in serum measured by ELISA (ElisaRSR™ GADAb) were 81.70 U/mL (normal <5 U/mL).

PAN disappeared readily after being started on IVIg. Cerebellar ataxia and tremor improved gradually after IVIg was administered monthly. Thrombocytopenia responded to high-dose IVIg. However, diabetes went unmodified under IVIg treatment. The patient was discharged, with the intention to repeat the IVIg treatment every 3 months at the dose described above. Right before the next IVIg administration (two and a half months after the previous one), the patient was only on clonazepam, and oscillopsia was present with recordings showing the reappearance of PAN with similar characteristics (Fig. 1C.3). Treatment with monthly IVIg was then scheduled, and the PAN disappeared again. After 1 year of follow-up, the patient remains asymptomatic for oscillopsia.

## Discussion and conclusions

This case report represents the first short cycle PAN successfully treated with IVIg after showing no response to a GABA_B_ agonist.

A few decades ago, oculomotor disturbances were found to account for the most prevalent sign of cerebellar and brainstem dysfunction in a large series of HIV-positive patients [6] and abnormal eye movements were present in 93% of HIV-infected patients with neurological signs [7]. Various nystagmus including central paroxysmal positional nystagmus [8] have been reported in HIV-positive individuals, but no case of PAN associated with HIV has been described previously.

Cerebellar symptoms in HIV-infected patients are thus frequent and might be the result of a direct effect of the virus [9, 10], an opportunistic infection such as the ones associated with JC virus [11, 12] and Cryptococcus [13], or a degeneration in the setting of an immune restoration disease related to the HIV infection [14]. No robust case of an HIV-positive patient with an immune-mediated cerebellar ataxia that responded to IVIg has ever been reported.

In addition to the positive response of PAN to IVIg, the development of other independent, autoimmune processes during the patient’s hospital admission might also suggest an autoimmune physiopathology for his PAN. Only one prior publication has been found to associate PAN with a specific antibody [4]. In that case report, the subject presented with progressive cerebellar ataxia and PAN along with positivity to GAD Ab measured by immunohistochemistry. The authors suggested that GAD Ab might have a pathogenic role, but PAN’s response to IVIg was not evaluated.

In our patient, we believe that the presence of GAD Ab measured by ELISA (ElisaRSR™ GADAb) was associated with the diagnosis of diabetes mellitus and not with the cerebellar syndrome. At the time of diagnosis of the cerebellar syndrome, GAD Ab measured by commercial ELISA kit (ElisaRSR™ GADAb) and by immunohistochemistry were both negative. Nowadays, immunohistochemistry has become the gold standard for the screening of onconeuronal, anti-neuropil and anti-GAD antibodies relevant to neurological diseases [5]. Only after diabetes mellitus appeared, GAD Ab measured by the same commercial ELISA kit was detected. We speculate that there may be another antibody yet to be elucidated that might be involved in our patient’s PAN and cerebellar ataxia.

It is well known that IVIg is successful in treating autoimmune diseases presumably mediated by antibodies, although its mechanism of action remains partly unclear. At high doses, IVIg might harbor anti-inflammatory effects.

To the best of our knowledge, there are isolated case reports of PAN caused by inflammatory diseases such as acute disseminated encephalomyelitis [15] and multiple sclerosis [16]. High-dose corticosteroid made PAN gradually disappear in light, but it persisted in darkness in the patient with multiple sclerosis. In the case of the patient with acute disseminated encephalomyelitis, three cycles of steroid pulse therapy and plasmapheresis followed by oral administration of a steroid and tacrolimus in addition to several types of anticonvulsants failed to resolve PAN. We propose that IVIg may have exerted its effect on our patient’s PAN through anti-idiotypic antibodies given that eminent anti-inflammatory medications such as steroids failed at treating PAN associated with inflammatory neurological conditions.

The elimination of PAN was not coincidental since it reappeared 3 months after the administration of the IVIg, and there was no recurrence after IVIg was given monthly. Therefore, this effect was reproducible and suggests that it might be worth trying IVIg where the clinical picture is suggestive of an autoimmune disorder.

## Abbreviations

CSF: Cerebrospinal fluid
ELISA: Enzyme-linked immunosorbent assay
GABA_B_: γ-Aminobutyric acid type B
GAD Ab: Glutamic acid decarboxylase 65 antibody
HEP: Horizontal eye position
HIV: Human immunodeficiency virus
HSPV: Horizontal slow-phase velocity
IVIg: Intravenous immunoglobulin
JC: John Cunningham
MRI: Magnetic resonance imaging
PAN: Periodic alternating nystagmus
PCR: Polymerase chain reaction
PET: Positron emission tomography
